# Effect of a conventional energy-restricted modified diet with or without meal replacement on weight loss and cardiometabolic risk profile in overweight women

**DOI:** 10.1186/1743-7075-8-64

**Published:** 2011-09-22

**Authors:** Christine E Metzner, Anke Folberth-Vögele, Norman Bitterlich, Martin Lemperle, Sandy Schäfer, Birgit Alteheld, Peter Stehle, Roswitha Siener

**Affiliations:** 1 Bonn Education Association for Dietetics r. A., Fürst-Pückler-Str. 44, D-50935 Cologne, Germany; 2 Department of Internal Medicine III, University Hospital, RWTH, Pauwelsstraße 44, D-52074 Aachen, Germany; 3 Ambulant Centre for Nutrition Education, Frankfurt am Main, Schweizer Straße 47, D- 60594, Frankfurt/M, Germany; 4 Medicine and Service Ltd, Department of Biostatistics, Boettcherstr. 10, D-09117 Chemnitz, Germany; 5 B-Vital, Ambulant Centre for Nutrition Education, Elsterwerdaer Platz 1, D-12683 Berlin, Germany; 6 Department of Nutrition and Food Sciences, Nutritional Physiology, University of Bonn, Endenicher Allee 11-13, AVZ1, D-53115 Bonn-Endenich, Germany; 7 Medical Nutrition Science, Department of Urology, University of Bonn, Sigmund-Freud-Straße 25, D-53105 Bonn, Germany

## Abstract

**Background:**

Abdominal obesity, atherogenic dyslipidemia and hypertension are essential risk factors for cardiovascular diseases. Several studies showed favorable effects of weight loss in overweight subjects on cardiometabolic risk profile.

**Methods:**

This open-label, randomized, controlled study investigated the effect of an energy-restricted modified diet with (MR) or without meal replacements for weight control (C) on weight loss, body composition and cardiometabolic risk profile in overweight women. Of 105 randomized participants, 87 were eligible for per protocol analysis. Anthropometric, clinical, blood, 24 h-urine parameters and dietary intake were assessed at baseline and after 12 weeks.

**Results:**

Dietary intervention resulted in a significant weight loss in both groups (MR: -5.98 ± 2.82 kg; p < 0.001, C: -4.84 ± 3.54 kg; p < 0.001). However, the rate of responder (weight loss >5%) was higher in MR (77%) versus C group (50%) (p = 0.010). A significant reduction in waist circumference (WC) and body fat mass (BFM) was observed in both groups. Body cell mass (BCM) and lean body mass (LBM) decreased, while percentage of BCM of body weight increased in MR more than in C group. Systolic and diastolic blood pressure (BP) significantly decreased and to a similar extent in both groups. Total cholesterol (TC), LDL-C but also HDL-C declined significantly in both groups, while no change occurred in triglycerides.

**Conclusions:**

Both dietary intervention strategies had a similar effect on weight loss and body fat distribution, but rate of responder was significantly higher in MR group. Systolic BP decreased to a similar extent in both groups. Cardiometabolic risk profile improved only partly in both groups.

## Introduction

The rising prevalence of obesity is considered a major cause for disorders such as type 2 diabetes (DM2) and cardiovascular diseases [1,2]. Type of body fat distribution seems to play a crucial role in the pathogenesis of obesity-related diseases. Due to its higher endocrine and metabolic activity compared to subcutaneous adipose tissue, visceral adipose tissue is regarded as a predictor of cardiometabolic risk factor levels [2-6]. Abdominal obesity is strongly correlated with visceral adipose tissue, clinically represented by the measurement of waist circumference (WC) [7]. A population-based cohort study of diabetes revealed that a larger waist circumference is associated with a higher risk of DM2, especially in women, whereas larger hip and thigh circumferences are clearly associated with a lower risk of diabetes [8].

The widespread lipid disorder in visceral obesity, insulin resistance and metabolic syndrome is atherogenic dyslipidemia, which is characterized by triad of elevated serum triglycerides (TG), low HDL-cholesterol (HDL-C) and small LDL-C particles [3]. Several studies showed favorable effects of visceral weight loss on serum TG in overweight subjects [4,9,10].

The question is whether the achieved weight loss alone is responsible for the reduction in blood pressure. Besides weight loss, mental stress reduction, dietary sodium restriction, and an increased intake of dietary flavanoids are suggested to reduce blood pressure [11-13]. Weight loss can be achieved by a conventional structured energy-restricted modified diet alone or in combination with meal replacements for weight control. Recent reports indicated that such meal replacements coupled with a low-calorie diet can offer an effective option for weight reduction and improvements in metabolic risk factors in overweight patients [14-16]. Therefore, aim of this prospective randomized study was to evaluate whether a diet with meal replacements (MR) can be as effective as a conventional energy-restricted modified diet (control diet, C) on weight loss, body composition and cardiometabolic risk profile in overweight women.

## Methods

### Participants

**Figure 1:**
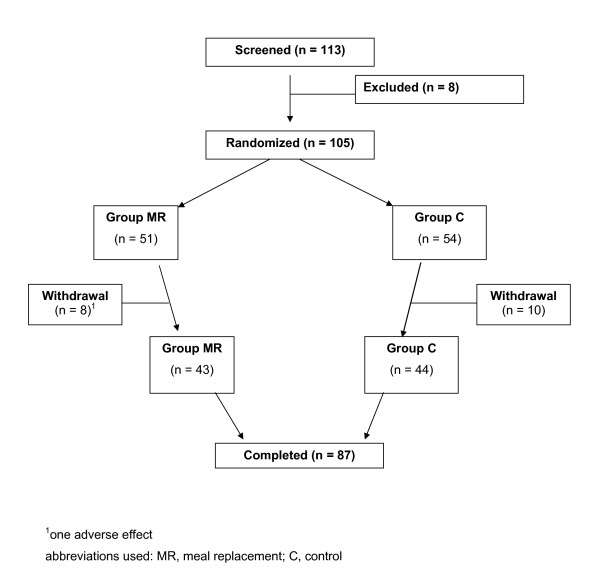
**Trial profile**.

Overweight women were recruited from two outpatient centers for nutrition education in Frankfurt/M and Berlin, Germany. Women aged between 18 and 60 years, BMI between 27.0 and 34.9 kg/m^2 ^and with one of the following blood lipids were included into the study: total cholesterol (TC) ≥ 200 mg/dL, LDL-cholesterol (LDL-C) ≥ 175 mg/dL, HDL-C ≤ 50 mg/dL, TG 150-400 mg/dL. Exclusion criteria were lactose or protein intolerance, hypo- or hyperthyroidism, pharmacological treatment of diabetes, intake of vitamins or mineral supplements, anticoagulants, cardiac pacemaker and contraindications to exercise. One hundred and thirteen subjects were screened (Figure 1). The study was approved by the ethics committees of the Chamber of Physicians of the German federal states of Hessen and Berlin. Informed consent was obtained from each participant prior to study onset.

### Study design

In a 12-week open-label, randomized, controlled intervention trial, subjects were randomly assigned to the MR or the C group, respectively, by a computer-generated identification number in order of recruitment. Blood samples, dietary records, clinical and biochemical data were collected at baseline and repeated 12 weeks after dietary intervention. Primary end point of the study was weight loss. The planned sample size of 44 subjects per group, excluding drop-out rate, was based on a power of 80% to detect a difference of at least 2.0 ± 3.0 kg (mean ± SD) between both groups at a level of significance of α = 0.05.

Both dietary intervention groups were instructed to follow an energy-restricted diet of approximately 1200 kcal/d. Three meals (breakfast, lunch, and dinner) were recommended. The food choice was based on vegetables, fruits, whole grain cereal and low-fat dairy products (1.5% fat). The MR group was advised to replace two meals, i.e. breakfast and dinner, every day with two MR shakes, soups or bars (FormMed HealthCare AG, Frankfurt/M, Germany) and to prepare their own lunch. The energy per serving of the MR meal is shown in Table 1. Each MR represented a balanced diet product for weight reduction according to article 14a of the dietary regulation issued by the German Federal Ministry of Food, Agriculture and Consumer Protection, based on Commission Directive 1996/8/EC of 26 February 1996. The C group was advised to follow a conventional energy-restricted modified diet with 15-20% of energy intake in the form of protein, 50-55% of energy intake in the form of carbohydrates and approximately 30% of energy intake in the form of fat according to previous studies [15,17].

**Table 1:** Daily dietary intake with meal replacements (per serving size)

	Shake	Soup	Bar
Energy (kcal)	205	200	218
Protein (g)	19	21.9	17.2
Carbohydrates (g)	16.9	12.9	27.0
Fat (g)	5.9	6.6	6.8
Fatty acids			
SAFA (g)	2.6	0.9	2.2
LA (g)	1.0	3.1	2.2
MUFA (g)	1.1	1.35	2.4
Dietary fibre (g)	2.4	1.5	3.1
			
**Minerals**			
Sodium (mg)	180	900	183
Potassium (mg)	510	605	539
Magnesium (mg)	200	61	82
Calcium (mg)	525	300	329
Phosphate (mg)	357	208	279
Iron (mg)	7.5	4.9	8
Zinc (mg)	5.0	3.0	7.4
Manganese (mg)	0.6	0.4	1.5
Copper (mg)	0.7	0.4	1.0
Iodine (μg)	75	41	75
Selenium (μg)	36	16.5	27
			
**Vitamins**			
Vitamin A (μg)	400	210	600
Vitamin B1 (mg)	0.7	0.3	0.8
Vitamin B2 (mg)	1.2	0.5	0.9
Vitamin B6 (mg)	1.0	0.5	1.1
Vitamin B12 (μg)	1.5	0.4	1.7
Vitamin C (mg)	45	13.5	34
Vitamin D (μg)	2.5	1.5	2.9
Vitamin E (mg)	10	3.0	11.5
Biotin (μg)	75	4.5	86
Folic acid (μg)	150	60	115
Nicotinic acid (mg)	12	5.4	10
Pantothenic acid (mg)	3.0	0.9	5.7

During a 12-week intervention period all participants attended ten group training sessions (eight to ten women per class) and four face-to-face visits for nutrition education. All participants received instruction manuals that included a sample meal plan, recipes and information regarding physical activity. Group training lasted one hour per session. Group training sessions were held separately for each group by a nutrition advisor. Topics of the structured sessions included practical knowledge of food, portion sizes and meal frequency with the aim of changing dietary behavior and lifestyle. The average duration of the monthly face-to-face visits was 25 minutes (range 10-40 minutes).

At baseline women were instructed on food selection, portion size estimation and accurate recording of dietary intakes. Three-day food records were documented at baseline and after the 12-week intervention. Dietary records were reviewed with each study participant and analyzed using PRODI 5.3 software (WVG, Stuttgart, Germany).

Body weight (kg) and height (cm) were measured. WC, HC and thigh circumference was determined via flexible tape. The goal to achieve > 5% weight loss was a targeted value. Body fat mass (BFM), body fat (%), body cell mass (BCM), and lean body mass (LBM) were determined by bioelectrical impedance analysis (BIA) with biaform^® ^software version 2.2 under standardized conditions [17]. Blood pressure (BP) was measured under standardized conditions after a 10 min. resting period. Anthropometric measurements and venous blood sampling were performed in the morning after an overnight fasting period of at least 12 h. Due to analysis of serum cortisol, blood samples were taken at 9:00 a.m. At baseline and after the 12-week dietary intervention, 24-hour urines were collected.

### Laboratory methods

Analyses of serum glucose (hexokinase method), uric acid (uricase-PAP method), gamma-glutamyltransferase (kinetic color test IFCC), total cholesterol (TC) (CHOD-PAP method), LDL-C and HDL-C (enzymatic color test), TG (GPO-PAP method) on Olympus 600 analyzer, homocysteine, folic acid and cortisol (chemiluminiscence method) on ADVIAR Centaur analyzer and glycated hemoglobin (HbA1c) (HPLC) on Biorad Variant II analyzer were conducted by the medical laboratory Potsdam, Germany.

Determination of the total antioxidative capacity (TAC) was performed by the reaction of antioxidants in the sample with a defined amount of exogenously provided hydrogen peroxide (H_2_O_2_). The antioxidants in the sample eliminate a certain amount of the provided hydrogen peroxide. The residual H_2_O_2 _is determined photometrically by an enzymatic reaction which involves the conversion of TMB to a colored product.

Analysis of 24 h urinary sodium excretion (ion sensitive electrode (ISE)) was performed at the laboratory of Medical Nutrition Science, Department of Urology, University of Bonn, Germany.

Laboratory quality certification was available for each parameter except for homocysteine, folic acid and TAC.

### Statistical methods

Statistical comparisons between groups were performed using the nonparametric Mann-Whitney U test for unpaired data. Pre-post intervention changes were analyzed via non-parametric Wilcoxon test. All statistical tests were two-sided. Differences were considered significant at p < 0.05. Data are reported as mean ± standard deviation (SD). ANCOVA including use of weight change and age as covariates was used to control for the potential confounder of systolic BP. Data analysis was performed based on the per protocol population and by using SPSS^® ^for Windows (version 19.0).

## Results

### Participants

A total of 105 overweight women were randomized, of whom 87 were eligible for per protocol (PP) analysis (Figure 1). Twelve women discontinued the study: one due to adverse effects (diarrhea) in the MR group and 11 for personal reasons (four women in the MR and seven women in the C group). Additionally, six women had to be excluded from analysis due to protocol violations. Meal replacements were well tolerated by the patients.

No differences were observed at baseline between both groups regarding anthropometric, clinical and biochemical parameters. Fifty-nine women (67.8%) had obesity stage I (Table 2). The ratio of obesity stage I (30.0-34.9 kg/m^2^) to preobesity (27.0-29.9 kg/m^2^) was approximately 2:1 in both groups. Abdominal adipose tissue distribution type (WC > 88 cm) did not differ between the MR and the C group. Systolic blood pressure (130/80 mmHg) was slightly above the cutoff in the MR group [3].

**Table 2:** Baseline characteristics of overweight women in the meal replacement (MR) and control group (C), respectively

	MR (n = 43) Mean ± SD	C (n = 44) Mean ± SD	MR vs C P
Age (years)	49.8 ± 11.7	49.6 ± 11.0	0.776^a^

Weight (kg)	83.9 ± 8.8	84.1 ± 8.3	0.946^a^

Height (cm)	163.8 ± 5.9	164.0 ± 5.8	0.909^a^

BMI (kg/m^2^)	31.2 ± 2.3	31.2 ± 2.1	0.912^a^
BMI 27.0 - 29.9 kg/m^2 ^(n)	14 (32.6%)	14 (31.8%)	0.941^b^
BMI 30.0 - 34.9 kg/m^2 ^(n)	29 (67.4%)	30 (68.2%)	0.941^b^

WC (cm)	93.9 ± 7.2	94.9 ± 6.1	0.491^a^
WC 80 - 88 cm (n)	12 (27.9%)	7 (15.9%)	0.176^b^
WC > 88 cm (n)	31 (72.1%)	37 (84.1%)	0.176^b^

BP systolic (mmHg)	131.2 ± 17.4	128.0 ± 19.7	0.185^a^
BP systolic ≥ 130 mmHg (n)	27 (62.8%)	22 (50.0%)	0.229^b^

BP diastolic (mmHg)	80.8 ± 9.6	79.8 ± 9.0	0.321^a^
BP diastolic ≥ 85 mmHg (n)	16 (37.2%)	11 (25.0%)	0.218^b^

Heart rate (1/min)	76.6 ± 10.6	77.8 ± 10.8	0.792^a^
Heart rate > 70/min (n)	33 (76.7%)	34 (77.3%)	0.953^b^

Oral contraceptives (n)	5 (11.6%)	3 (6.8%)	0.438^b^

Smokers (n)	7 (16.3%)	9 (20.5%)	0.615^b^

abbreviations used: BMI, body mass index; BP, blood pressure; WC, waist circumference

^a^Mann-Whitney-U-test

^b^Chi^2^-Test

### Anthropometric and clinical characteristics

Dietary intervention resulted in a significant weight loss in both groups, without significant difference between the MR and the C group (Table 3). However, relative weight loss in the MR group was higher compared to the C group (p = 0.048). Moreover, the rate of responder (>5% weight loss) was higher in the MR group (33 women, 77%) versus the C group (22 women, 50%) after the 12-week dietary intervention (p = 0.010) (Table 4). While women with WC ≤ 88 cm (21.8% in both groups) lost significantly more weight in the MR group (MR: - 5.98 ± 2.28 kg, C: - 0.96 ± 2.29 kg; p = 0.002), no significant difference in weight change was observed in women with WC > 88 cm (MR: -5.98 ± 3.04 kg, C: - 5.57 ± 3.25 kg; p = 0.506).

**Table 3:** Anthropometric and clinical characteristics in overweight women at baseline and after dietary intervention

	MR (n = 43) Mean ± SD			C (n = 44) Mean ± SD			MR vs C
	**Baseline**	**Week 12**	**Difference**	**Baseline**	**Week 12**	**Difference**	**P^a^**

Weight (kg)	83.9 ± 8.8	78.0 ± 9.2**	-5.98 ± 2.82	84.1 ± 8.3	79.3 ± 7.3**	-4.84 ± 3.54	0.075

Relative weight loss (%)	100 ± 0	92.8 ± 3.3**	-7.19 ± 3.25	100 ± 0	94.4 ± 4**	-5.60 ± 4.02	0.048

BMI (kg/m^2^)	31.2 ± 2.3	29.0 ± 2.6**	-2.24 ± 1.02	31.2 ± 2.1	29.4 ± 1.9**	-1.79 ± 1.31	0.075

WC (cm)	93.9 ± 7.2	87.4 ± 7.6**	-6.55 ± 3.91	94.9 ± 6.1	89.6 ± 6.3**	-5.30 4.27	0.094

WHtR	0.574 ± 0.043	0.534 ± 0.047**	0.040 ± 0.024	0.579 ± 0.040	0.547 ± 0.045**	0.032 ± 0.026	0.092

HC (cm)^b^	114.2 ± 6.4 (n = 26)	108.5 ± 6.0	-5.71 ± 2.34	114.3 ± 6.0 (n = 28)	109.4 ± 6.5	-4.93 ± 3.45	0.565

WHR^b^	0.83 ± 0.06 (n = 26)	0.82 ± 0.05	-0.01 ± 0.03	0.84 ± 0.05 (n = 28)	0.84 ± 0.06	0.00 ± 0.03	0.350

Thigh circumference (cm)^b^	64.1 ± 5.0	60.8 ± 5.1	-3.33 ± 2.10	64.1 ± 4.6	61.5 ± 5.0	-2.59 ± 2.35	0.158
	(n = 35)			(n = 38)			

BFM (kg)	36.1 ± 6.3	31.4 ± 6.4**	-4.69 ± 2.31	36.6 ± 6.0	32.6 ± 5,2**	-3.98 ± 2.53	0.144

Body fat (%)	42.7 ± 3.8	39.9 ± 4.3**	-2.79 ± 1.79	43.3 ± 3.6	41.0 ± 3.6*	-2.33 ± 1.51	0.191

BCM (kg)	25.7 ± 2.5	24.8 ± 2.6**	-0.93 ± 0.80	25.7 ± 2.3	24.7 ± 2.0**	-0.98 ± 0.98	0.760

BCM (%)	30.8 ± 2.9	32.0 ± 3.1**	1.19 ± 1.24	30.7 ± 3.0	31.3 ± 2.9*	0.62 ± 1.17	0.054

LBM (kg)	48.1 ± 3.8	46.8 ± 3.9**	-1.30 ± 1.40	47.7 ± 3.8	46.9 ± 3.3**	-0.77 ± 1.46	0.058

LBM (%)	57.6 ± 3.8	60.4 ± 4.3**	2.81 ± 2.22	56.9 ± 3.7	59.4 ± 3.7**	2.45 ± 1.93	0.445

BP systolic (mmHg)	131.2	119.8	-11.4	128.0	119.5	-8.5	0.148
	± 17.4	± 12.8**	± 18.8	± 19.7	± 13.8*	± 23.5	

BP diastolic (mmHg)	80.8 ± 9.6	77.2 ± 8.6*	-3.6 ± 11.7	79.8 ± 9.0	75.5 ± 8.3*	-4.4 ± 11.7	0.914

Heart rate (1/min)^b^	77.0 ± 10.3 (n = 42)	75.8 ± 9.9	-1.2 ± 8.0	77.8 ± 10.8	75.8 ± 11.5	-2.0 ± 8.9	0.983

abbreviations used: BCM, body cell mass; BFM, body fat mass; BMI, body mass index; BP, blood pressure; C, control group; HC, hip circumference; LBM, lean body mass; MR, meal replacement group; WC, waist circumference; WHR, Waist-to-hip ratio; WHtR, Waist-to-height ratio

*p < 0.05; ** p < 0.001; Wilcoxon-test within groups

^a^p-value: Mann-Whitney-U-test

^b^data not available for all women

**Table 4:** Change in the cardiometabolic risk profile in overweight women after dietary intervention

	MR (n = 43) n (%)			C (n = 44) n (%)			MR vs C
	**Baseline**	**Week 12**	**P^a^**	**Baseline**	**Week 12**	**P^a^**	**P^b^**

Weight reduction > 5%	-	33 (76.7%)	-	-	22 (50.0%)	-	0.010

WC > 88 cm	31 (72.9%)	19 (44.2%)	0.009	37 (84.1%)	24 (54.5%)	0.003	0.429

BP systolic ≥ 130 mmHg	28 (65.1%)	15 (34.9%)	0.005	20 (45.5%)	12 (27.3%)	0.076	0.263

BP diastolic ≥ 85 mmHg	13 (30.2%)	9 (20.9%)	0.323	6 (13.6%)	6 (13.6%)	1.000	0.229

BP ≥ 130/85 mmHg	12 (27.9%)	5 (11.6%)	0.058	6 (13.6%)	5 (11.4%)	0.747	0.439

TG ≥ 150 mg/dl	14 (41.2%)^c^	16 (47.1%)^c^	0.651	15 (44.1%)^c^	13 (38.2%)^c^	0.647	0.897

HDL-C < 50 mg/dl	11 (32.4%)^c^	12 (35.3%)^c^	0.808	8 (23.5%)^c^	11 (32.4%)^c^	0.437	0.851

Fasting glucose ≥ 110 mg/dl	2 (5.9%)^c^	2 (5.9%)^c^	1.000	3 (8.8%)^c^	1 (2.9%)^c^	0.306	0.906

Hypertriglyceridemic waist^d^	12 (35.3%)^c^	7 (20.6%)^c^	0.194	14 (41.2%)^c^	7 (20.6)^c^	0.080	0.984

Atherogenic dyslipidemia^e^	6 (17.6%)^c^	7 (20.6%)^c^	0.763	4 (11.8%)^c^	3 (8.8%)^c^	0.694	0.490

abbreviations used: BP, blood pressure; C, control group; HDL-C, HDL cholesterol; MR, meal replacement group; WC, waist circumference

^a^p-value: chi-square-test within groups

^b^p-value: chi-square-test in contingency tables

^c^biochemical analysis with the same method (n = 34)

^d^hypertriglyceridemic waist, i.e. WC > 88 cm and TG ≥ 150 mg/dL [18]

^e^atherogenic dyslipidemia, i.e. triad of elevated triglycerides, low HDL-C, and small LDL-C particles [3]

Dietary intervention significantly reduced mean BMI from obesity stage I to preobesity in both groups (Table 3). Correspondingly, WC and waist-to-height ratio (WHtR) significantly decreased to a similar extent. After the 12-week dietary intervention, mean WC was below the cutoff of 88 cm only in the MR group [3]. Within group analysis revealed a significant reduction in BFM, body fat (%), BCM, and LBM in both groups. After the 12-week dietary intervention, mean systolic and diastolic BP were within the normal range.

### Clinical chemistry and biochemical characteristics

After the 12-week dietary intervention TC, LDL-C but also HDL-C significantly decreased in both groups, without significant differences between the groups (Table 5). No change was observed in serum TG, glucose and uric acid within and between groups during intervention. Serum homocysteine concentration increased significantly only in the C group, but this was without clinical relevance. Serum folic acid concentration increased significantly only in the MR group. Serum cortisol concentration and TAC remained unchanged during the intervention period. GGT activity decreased significantly in both groups to a similar extent.

**Table 5:** Clinical chemistry and biochemical characteristics in overweight women at baseline and after dietary intervention

	**MR (n = 34) ^a ^Mean ± SD**			C (n = 34) ^a ^Mean ± SD			MR vs C
	**Baseline**	**Week 12**	**Difference**	**Baseline**	**Week 12**	**Difference**	**P^b^**

Glucose (mg/dL)	91.8 ± 13.7	92.4 ± 10.9	0.63 ± 10.94	90.7 ± 14.3	88.1 ± 13.1	-2.66 ± 10.74	0.315

HbA1c (%)	5.54 ± 0.63	5.49 ± 0.47	-0.05 ± 0.64	5.63 ± 0.51	5.46 ± 0.48	-0.16 ± 0.61	0.201

TC (mg/dL)	241.2 ± 53.1	224.4 ± 53.2*	-16.7 ± 33.7	241.5 ± 34.3	230.3 ± 40.9*	-11.2 ± 25.9	0.556

HDL-C (mg/dL)	59.1 ± 16.1	55.3 ± 14.1**	-3.7 ± 6.2	59.2 ± 11.5	55.7 ± 10.9*	-3.5 ± 5.2	0.864

LDL-C (mg/dL)	157.0 ± 39.7	144.0 ± 38.4**	-13.0 ± 18.2	161.4 ± 24.2	152.3 ± 30.8*	-9.1 ± 18.7	0.647

TG (mg/dL)	186.4 ± 145.6	161.1 ± 108.0	-25.3 ± 110.4	152.5 ± 83.8	141.6 ± 66.1	-10.9 ± 52.9	0.861

TG/HDL-C	3.59 ± 3.55	3.19 ± 2.49	-0.40 ± 2.44	2.71 ± 1.66	2.62 ± 1.37	-0.09 ± 0.96	0.880

LDL-C/HLD-C	2.81 ± 0.86	2.73 ± 0.86	-0.08 ± 0.33	2.81 ± 0.63	2.79 ± 0.68	-0.02 ± 0.33	0.539

GGT (U/L)	33.5 ± 28.8	27.8 ± 20.1	-5.72 ± 12.91*	27.9 ± 13.3	24.0 ± 10.6	-3.85 ± 4.92**	0.759

Uric acid (mg/dL)	4.82 ± 1.18	4.59 ± 0.94	-0.23 ± 0.70	4.86 ± 1.01	4.75 ± 0.97	0.11 ± 0.66	0.384

Homocysteine (μmol/L)	11.7 ± 4.1	11.0 ± 2.7	-0.63 ± 2.66	10.3 ± 2.0	11.7 ± 2.7*	1.36 ± 2.65	0.007

Folic acid (μg/L)	10.6 ± 4.9	15.2 ± 5.8**	4.62 ± 5.74	11.1 ± 4.3	12.3 ± 7.2	1.17 ± 6.28	0.005

GFR (mL/min/1.73 m^2^)	73.6 ± 12.0	73.3 ± 12.1	-0.24 ± 6.50	74.8 ± 8.9	72.5 ± 8.8	-2.27 ± 6.10	0.202

TAC (μmol/L)	266.2 ± 30.8	276.6 ± 38.4	10.4 ± 45.6	280.9 ± 43.0	282.2 ± 45.7	1.3 ± 48.5	0.210

Cortisol (nmol/L)	424.3 ± 142.5	532.1 ± 179.6	107.8 ± 145.6	387.2 ± 164.6	477.4 ± 140.9	90.2 ± 176.0	0.826

TAC/Cortisol (10^3^)	0.70 ± 0.27	0.58 ± 0.20	-0.12 ± 0.27	0.89 ± 0.49	0.65 ± 0.25	-0.24 ± 0.41	0.498

U-sodium (mmol/24 h)^c^	162.2 ± 64.5	158.2 ± 94,1	-4.0 ± 118.0	158.3 ± 68.1	170.8 ± 81.2	12.5 ± 82.9	0.283

abbreviations used: GFR, glomerular filtration rate; GGT, gamma-glutamyltransferase; HbA1c, glycated haemoglobin; HDL-C, HDL cholesterol; LDL-C, LDL cholesterol; TAC, total antioxidative capacity; TC, total cholesterol; TG, triglycerides; U-sodium, urinary sodium

^a^biochemical measurements with the same method

^b^p-value: Mann-Whitney-U-test; Wilcoxon-test within groups: *p < 0.05; **p < 0.001

^c^n = 26 in the MR group and n = 28 in the C group

### Cardiometabolic risk profile

Table 4 reflects results related to the cardiometabolic risk profile. The total number of subjects classified with atherogenic dyslipidemia [3] did not reduce significantly during the study. After the 12-week dietary intervention hypertriglyceridemic waist phenotype, i.e. WC > 88 cm and TG ≥ 150 mg/dL [18], decreased by 42% in the MR group and by 50% in the C group.

### Dietary intake

At baseline, the reported energy intake was similar in both groups (MR: 1574 ± 408 kcal/d; C: 1683 ± 414 kcal/d). After the 12-week intervention energy intake was significantly reduced within each group but not between both groups (MR: 1268 ± 306 kcal/d; C: 1406 ± 392 kcal/d). The calculated energy expenditure estimated with Harris-Benedict equation [19] at a physical activity level (PAL) of 1.6 differed from these data in both groups at baseline (MR group: 2356 ± 191 kcal/d C group: 2378 ± 163 kcal/d) and therefore suggested underreporting. Dietary sodium intake was estimated from 24 h urinary sodium excretion.

## Discussion

Our results indicate that both weight loss strategies are effective treatment options for overweight women. The goal to achieve a weight loss of more than 5% (4.2 kg of mean body weight at baseline) after 12 weeks was exceeded by at least 1.8 kg in the MR group but only by 0.6 kg in the C group, which is reflected in the significantly increased responder rate of the MR group. These results can be attributed to the preference for convenience foods [20] The weight loss of approximately 5% achieved in both groups is similar to that reported for preobesity and obesity stage I [14-16,21]. It has been reported that especially time and structure of the nutritional education play an important role in achieving high compliance [14]. Moreover, regular education to achieve an increase in physical activity is necessary, especially for maintaining the body weight [22].

For the evaluation of cardiometabolic risk not only body weight is important. The role of the body fat distribution phenotype has been shown to be even more prominent than that of BMI [23]. Compared to visceral fat, a high proportion of subcutaneous fatty tissue is accompanied by a lower cardiometabolic risk. Therefore, reduction in WC is considered to be more essential than loss of body weight alone, especially in DM2 [24]. Studies have shown that WHtR is the best indicator of cardiovascular risk in different measures of abdominal obesity and that it can be used to assess body composition [25]. In the present study, the significant decrease in WC and WHtR in both groups during dietary intervention suggests that the body fat distribution phenotype has changed. A general cutoff of 0.5 has been suggested for WHtR [26]. However, this value was not achieved in our study population.

A strong association has been demonstrated between WC and plasma TG [27]. After the 12-week dietary intervention a decrease in WC by 6.6 cm in the MR group and by 5.5 cm in the C group was achieved. Surprisingly, in our study no significant reduction in fasting serum TG and glucose was observed in the present trial.

In our study, a similar reduction of TC and LDL-C was found in both groups, which can be attributed to energy restriction and the resulting weight loss. Although the significant decrease in HDL-C is remarkable, mean HDL-C at baseline and after the 12-week dietary intervention was above the cutoff of 50 mg/dL. It is notable that the number of women with a HDL-C below 50 mg/dL, TG above 150 mg/dL and atherogenic dyslipidemia, respectively, did not change in both groups. Although several studies found that weight reduction in overweight and obese subjects favorably modifies atherogenic dyslipidemia [28,29], we were unable to confirm these findings. One reason could be the fatty acid composition of the dietary intervention with an insufficient proportion of MUFA and especially long-chain n-3 polyunsaturated fatty acids (n-3 LC PUFA).

Moreover, the 12-week dietary intervention resulted in a significant and similar improvement of systolic and diastolic BP in both groups. However, the number of women with systolic BP ≥ 130 mmHg decreased significantly only in the MR group and the number of women with a BP of 130/85 mmHg tended to decrease after 12 weeks.

In the present study, body weight loss resulted in a significant reduction in systolic BP in the study population (p = 0.002) (Table 6). This effect depended on the body weight at baseline. The higher the initial body weight, the higher was the reduction in systolic BP and diastolic BP. One kg of weight loss resulted in a reduction of systolic and diastolic BP by 2.0 and 1.1 mmHg, respectively. The effect of weight loss on systolic BP was more pronounced in the C group and amounted to 3.5 mmHg per kg body weight (p = 0.001) as compared to 1.0 mmHg per kg body weight in the MR group (p = 0.480). Diastolic BP was similar with 1.7 mmHg per kg body weight in the C group (p < 0.001), and 0.4 mmHg in the MR group (p = 0.545).

**Table 6:** Systolic blood pressure depending on body weight, sodium intake and serum cortisol (age-adjusted)

Body weight			
**Group**	**Difference**	**RC B**	**P**
C (n = 34)	-5.0 ± 3.5	3.516	0.001
MR (n = 33)	-6.6 ± 2.6	1.028	0.480

**Urinary sodium**			

Group	Difference	RC B	P
C (n = 34)	3.5 ± 70.6	-0.051	0.383
MR (n = 33)	-29.7 ± 86.7	0.068	0.106

**Serum cortisol**			

Group	Difference	RC B	P
C (n = 34)	95.2 ± 164.6	0.041	0.105
MR (n = 33)	118.7 ± 159.1	-0.015	0.515

abbreviations used: C, control group; MR, meal replacement group; RC B, regression coefficient B

Apart from the influence of weight loss on the systolic blood pressure, dietary sodium intake, estimated from 24 h urinary sodium excretion, seemed to exert different effects on both groups. No convincing association was found between systolic BP and serum cortisol and between systolic BP and sodium intake (Table 6). Body cell mass (BCM) and lean body mass (LBM) decreased in both groups, while the percentage of BCM of body weight increased more in the MR group than in the C group. The higher increase in the difference in absolute BCM in the MR group versus C group resulted in a reduction in systolic BP, whereas BCM affected systolic BP in the C group only via weight reduction (Table 6 and 7). The different changes in body composition and the different impact of body weight are reflected in non-BCM and BCM. The result from the C group shows that non-BCM is the dominant factor for systolic BP reduction, while BCM was found to be the major factor in the MR group. The systolic BP decreased to a similar extent in both groups despite different influencing variables.

**Table 7:** Systolic blood pressure depending on BCM and non-BCM (age-adjusted)

Group	P ANCOVA	dnon-BCM	dBCM
Total	0.005	2.257 (0.7 - 3.8) 0.004	0.135 (-4.8 - 5.1) 0.957

C	0.013	2.765 (0.5 - 5.0) 0.018	3.226 (-4.0 - 10.4) 0.372

MR	0.218	1.231 (-1.0 - 3.5) 0.278	-4.689 (-11.9 - 2.6) 0.198

abbreviations used: BCM, body cell mass; C, control group; d, difference; MR, meal replacement group

GGT is the enzyme responsible for initiating extracellular catabolism of glutathione [30]. Increased GGT activity may be a response to oxidative stress, which can increase the transport of glutathione precursors into cells [30,31] and is involved in the generation of reactive oxygen species [31]. Therefore, it has been suggested that higher GGT levels are associated with an increased risk for the development of hypertension [32]. In our study, we found a significant reduction in GGT activity in both groups.

Evidence suggests that reactive oxygen species are fundamentally involved in hyperhomocysteinemia, which, together with a low folate status, is a major risk factor for atherosclerosis [33]. In the present study, a significant higher serum homocysteine was found after the 12-week dietary intervention in the C group when compared to the MR group. This result was probably due to a better folate status through increased intake with the MR, as demonstrated by the significant increase in serum folic acid in the MR group.

In conclusion, both groups exhibited an effective weight loss. Compliance was higher in the MR group than in the C group as demonstrated by the higher responder rate in the MR (77%) than in the C group (50%). Cardiometabolic risk was positively affected mainly through the decrease in BP, TC and LDL-C, whereas other risk factors were not affected by the 12-week dietary intervention. Systolic BP decreased to a similar extent in both groups despite different influencing variables. The significant lower serum homocysteine and higher folic acid after the 12-week dietary intervention in the MR group was probably due to a better folate status through increased intake with the MR. Cardiometabolic risk profile improved only partly in both groups.

## Competing interests

The authors declare that they have no competing interests.

## Authors' contributions

CM was the scientific project manager and acted as an expert on metabolic syndrome. ML conducted the study (supervisor). AFV and SS were responsible for patient inclusion and conducted the study. RS was responsible for the study design and contributed to the interpretation of the data. NB conducted the statistical analysis. CM, RS, BA and AFV constituted the writing group and prepared the paper. ML and PS revised the manuscript. This study was supported in part by a research grant from FormMed HealthCare AG, Frankfurt, Germany. ML is CEO, CM is a consultant and AFV was an employee of FormMed HealthCare AG, Frankfurt, Germany. All authors contributed to the final version of the paper and gave their approval for publication of the final version.

## References

[B1] Bray GA Medical consequences of obesity J Clin Endocrinol Metab 2004 89 2583 2589 10.1210/jc.2004-0535 15181027

[B2] Després JP Lemieux I Bergeron J Pibarot P Mathieu P Larose E Rodés-Cabau J Bertrand OF Poirier P Abdominal obesity and the metabolic syndrome: contribution to global cardio-metabolic risk Arterioscler Thromb Vasc Biol 2008 28 1039 1049 10.1161/ATVBAHA.107.159228 18356555

[B3] Third report of the National Cholesterol Education Program (NCEP) Expert Panel on Detection, Evaluation, and Treatment of High Blood Cholesterol in Adults (Adult Treatment Panel III) Final Report Circulation 2002 106 3143 3421 12485966

[B4] Kahn HS Valdez R Metabolic risks identified by the combination of enlarged waist and elevated triacylglycerol concentration Am J Clin Nutr 2003 78 928 934 14594778 10.1093/ajcn/78.5.928

[B5] Bray GA Jablonski KA Fujimoto WY Barrett-Connor E Haffner S Hanson RL Hill JO Hubbard V Kriska A Stamm E Pi-Sunyer FX Diabetes Prevention Program Research Group Relation of central adiposity and body mass index to the development of diabetes in the Diabetes Prevention Program Am J Clin Nutr 2008 87 1212 1218 18469241 10.1093/ajcn/87.5.1212 PMC2517222

[B6] Demerath EW Reed D Rogers N Sun SS Lee M Choh AC Couch W Czerwinski SA Chumlea WC Siervogel RM Towne B Visceral adiposity and its anatomical distribution as predictors of the metabolic syndrome and cardiometabolic risk factor levels Am J Clin Nutr 2008 88 1263 1271 18996861 10.3945/ajcn.2008.26546 PMC2801427

[B7] Jacobs EJ Newton CC Wang Y Patel AV McCullough ML Campbell PT Thun MJ Gapstur SM Waist circumference and all-cause mortality in a large US cohort Arch Intern Med 2010 170 1293 1301 10.1001/archinternmed.2010.201 20696950

[B8] Snijder MB Dekker JM Visser M Bouter LM Stehouwer CDA Kostense PJ Yudkin JS Heine RJ Nijpels G Seidell JC Association of hip and thigh circumferences independent of waist circumference with the incidence of type 2 diabetes: the Hoorn Study Am J Clin Nutr 2003 77 1192 1197 12716671 10.1093/ajcn/77.5.1192

[B9] Park HS Lee K Greater beneficial effects of visceral fat reduction compared with subcutaneous fat reduction on parameters of the metabolic syndrome: a study of weight reduction programmes in subjects with visceral and subcutaneous obesity Diabet Med 2005 22 266 272 10.1111/j.1464-5491.2004.01395.x 15717873

[B10] Lee JW Lee HR Shim JY Im JA Lee DC Abdominal visceral fat reduction is associated with favorable changes of serum retinol binding protein-4 in nondiabetic subjects Endocr J 2008 55 811 818 10.1507/endocrj.K08E-030 18493106

[B11] Björntorp P Do stress reactions cause abdominal obesity and comorbidities? Obesity Rev 2001 2 73 86 10.1046/j.1467-789x.2001.00027.x 12119665

[B12] Ard JD Coffman CJ Lin PH Svetkey LP One-year follow-up study of blood pressure and dietary patterns in dietary approaches to stop hypertension (DASH)-sodium participants Am J Hypertens 2004 17 1156 1162 10.1016/j.amjhyper.2004.07.005 15607623

[B13] Taubert D Roesen R Lehmann C Jung N Schömig E Effects of low habitual cocoa intake on blood pressure and bioactive nitric oxide: a randomized controlled trial JAMA 2007 298 49 60 10.1001/jama.298.1.49 17609490

[B14] Ashley JM St Jeor ST Perumean-Chaney S Schrage J Bovee V Meal replacements in weight intervention Obes Res 2001 9 Suppl 4 312S 320S 11707559 10.1038/oby.2001.136

[B15] Flechtner-Mors M Ditschuneit HH Johnson TD Suchard MA Adler G Metabolic and weight loss effects of long-term dietary intervention in obese patients: four-year results Obes Res 2000 8 399 402 10.1038/oby.2000.48 10968732

[B16] Heymsfield SB van Mierlo CAJ van der Knaap HCM Heo M Frier HI Weight management using a meal replacement strategy: meta and pooling analysis from six studies Int J Obes 2003 27 537 549 10.1038/sj.ijo.0802258 12704397

[B17] Kyle UG Bosaeus I De Lorenzo AD Deurenberg P Elia M Gómez JM Heitmann BL Kent-Smith L Melchior JC Pirlich M Scharfetter H Schols AMWJ Pichard C Bioelectrical impedance analysis--part II: utilization in clinical practice Clin Nutr 2004 23 1430 1453 10.1016/j.clnu.2004.09.012 15556267

[B18] LaMonte MJ Ainsworth BE DuBose KD Grandjean PW Davis PG Yanowitz FG Durstine JL The hypertriglyceridemic waist phenotype among women Atherosclerosis 2003 171 123 130 10.1016/j.atherosclerosis.2003.07.008 14642414

[B19] Frankenfield DC Muth ER Rowe WA The Harris-Benedict studies of human basal metabolism: history and limitations J Am Diet Assoc 1998 98 439 445 10.1016/S0002-8223(98)00100-X 9550168

[B20] Noakes M Foster PR Keogh JB Clifton PM Meal replacements are as effective as structured weight-loss diets for treating obesity in adults with features of metabolic syndrome J Nutr 2004 134 1894 1899 15284372 10.1093/jn/134.8.1894

[B21] König D Deibert P Frey I Landmann U Berg A Effect of meal replacement on metabolic risk factors in overweight and obese subjects Ann Nutr Metab 2008 52 74 78 10.1159/000119416 18319587

[B22] Klein S Sheard NF Pi-Sunyer X Daly A Wylie-Rosett J Kulkarni K Clark NG Weight management through lifestyle modification for the prevention and management of type 2 diabetes: rationale and strategies: A statement of the American Diabetes Association, the North American Association for the Study of Obesity, and the American Society for Clinical Nutrition Diabetes Care 2004 27 2067 2073 10.2337/diacare.27.8.2067 15277443

[B23] Lofgren I Herron K Zern T West K Patalay M Shachter NS Koo SI Fernandez ML Waist circumference is a better predictor than body mass index of coronary heart disease risk in overweight premenopausal women J Nutr 2004 134 1071 1076 15113947 10.1093/jn/134.5.1071

[B24] Tankó LB Bagger YZ Qin G Alexandersen P Larsen PJ Christiansen C Enlarged waist combined with elevated triglycerides is a strong predictor of accelerated atherogenesis and related cardiovascular mortality in postmenopausal women Circulation 2005 111 1883 1890 10.1161/01.CIR.0000161801.65408.8D 15837940

[B25] Schneider HJ Friedrich N Klotsche J Pieper L Nauck M John U Dörr M Felix S Lehnert H Pittrow D Silber S Völzke H Stalla GK Wallaschofski H Wittchen HU The predictive value of different measures of obesity for incident cardiovascular events and mortality J Clin Endocrinol Metab 2010 95 1777 1785 10.1210/jc.2009-1584 20130075

[B26] Ashwell M Hsieh SD Six reasons why the waist-to-height ratio is a rapid and effective global indicator for health risks of obesity and how its use could simplify the international public health message on obesity Int J Food Sci Nutr 2005 56 303 307 10.1080/09637480500195066 16236591

[B27] Lemieux I Pascot A Couillard C Lamarche B Tchernof A Alméras N Bergeron J Gaudet D Tremblay G Prud'homme D Nadeau A Despres JP Hypertriglyceridemic waist: A marker of the atherogenic metabolic triad (hyperinsulinemia; hyperapolipoprotein B; small, dense LDL) in men? Circulation 2000 102 179 184 10889128 10.1161/01.cir.102.2.179

[B28] National Institutes of Health Clinical guidelines on the identification, evaluation, and treatment of overweight and obesity in adults - the evidence report 1998 NIH Pub No 98-4083. Bethesda, MD: National Heart, Lung and Blood Institute 228 9813653

[B29] National Institutes of Health Clinical guidelines on the identification, evaluation, and treatment of overweight and obesity in adults - the evidence report Obesity Res 1998 6 Suppl 2 51 209 9813653

[B30] Whitfield JB Gamma glutamyl transferase Crit Rev Clin Lab Sci 2001 38 263 355 10.1080/20014091084227 11563810

[B31] Lee DH Blomhoff R Jacobs DR Is serum gamma glutamyltransferase a marker of oxidative stress? Free Radic Res 2004 38 535 539 10.1080/10715760410001694026 15346644

[B32] Sabanayagam C Shankar A Li J Pollard C Ducatman A Serum gamma-glutamyl transferase level and diabetes mellitus among US adults Eur J Epidemiol 2009 24 369 373 10.1007/s10654-009-9346-7 19449164

[B33] Stanger O Weger M Interactions of homocysteine, nitric oxide, folate and radicals in the progressively damaged endothelium Clin Chem Lab Med 2003 41 1444 1454 10.1515/CCLM.2003.222 14656024

